# Biodegradable Fiber-Reinforced Gluten Biocomposites for Replacement of Fossil-Based Plastics

**DOI:** 10.1021/acsomega.3c07711

**Published:** 2023-12-01

**Authors:** Antonio J. Capezza, Mercedes Bettelli, Xinfeng Wei, Mercedes Jiménez-Rosado, Antonio Guerrero, Mikael Hedenqvist

**Affiliations:** † Department of Fibre and Polymer Technology, KTH Royal Institute of Technology, Teknikringen 56, Stockholm SE-100 44, Sweden; ‡ Department of Chemical Engineering, Universidad de Sevilla, Sevilla 41012, Spain

## Abstract

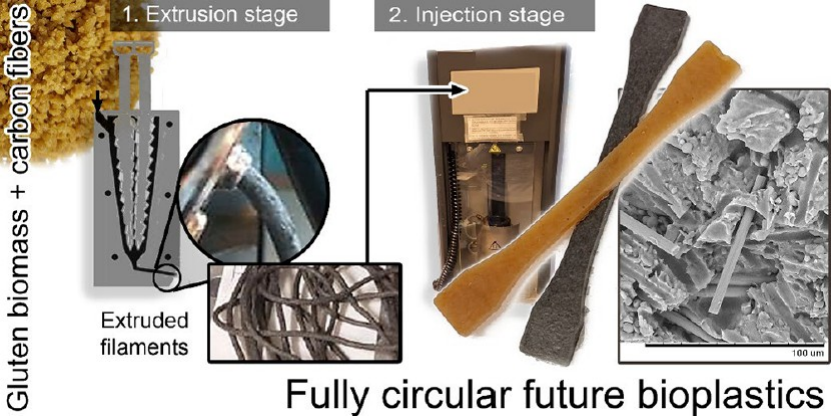

Biocomposites based on wheat gluten and reinforced with carbon fibers were produced in line with the strive to replace fossil-based plastics with microplastic-free alternatives with competing mechanical properties. The materials were first extruded/compounded and then successfully injection molded, making the setup adequate for the current industrial processing of composite plastics. Furthermore, the materials were manufactured at very low extrusion and injection temperatures (70 and 140 °C, respectively), saving energy compared to the compounding of commodity plastics. The sole addition of 10 vol % fibers increased yield strength and stiffness by a factor of 2–4 with good adhesion to the protein. The biocomposites were also shown to be biodegradable, lixiviating into innocuous molecules for nature, which is the next step in the development of sustainable bioplastics. The results show that an industrial protein coproduct reinforced with strong fibers can be processed using common plastic processing techniques. The enhanced mechanical performance of the reinforced protein-based matrix herein also contributes to research addressing the production of safe materials with properties matching those of traditional fossil-based plastics.

## 1 Introduction

Bioplastic processing is a critical component in studies dealing with developing sustainable future plastics for ensuring their successful implementation in current industrial production lines.^1,2^ The mechanical and processing techniques of bioplastics must match that of synthetic plastics, including polyolefins such as polyethylene (PE) and polypropylene (PP).^3^ The possibility of implementing bioplastic production and replacing synthetic counterparts is also related to the availability of raw materials, ensuring that the expected demand of ca. 600 million metric tons of plastic products in 2026 is met.^4^ In this regard, proteins obtained from agro-food industries as coproducts are envisioned as a potential candidate to fulfill the demand for bioplastics with matching mechanical properties to synthetic options.^5^

Wheat gluten (WG), a coproduct from the wheat starch industry, e.g., for bioethanol production, has been demonstrated as a versatile biopolymer that can be thermally processed into various products, ranging from stiff foams to superabsorbent particles.^6−10^ Moreover, the properties of the gluten-based bioplastics were also demonstrated to be readily tuned by adding fillers/fiber mats to increase their mechanical strength (reaching up to 50 MPa) or changing their electrical conductivity toward conductive biofoams.^5,9^ Several polymer processing techniques have been tested to produce WG-based materials with the desired shapes. An advantage of using a protein-based matrix, especially WG, is that the product’s properties can be readily tuned by the sole modification of the mixture (such as a pH change) and/or varying the processing temperature.^11−15^ The next challenge for WG-based bioplastics is to be able to match the mechanical performance of commercial polyolefins in applications where injection molding is used to enable high-volume and cost-effective production of plastic components of both simple and complex shapes at different size levels (everything from car bumpers to millimeter/centimeter-sized parts in, e.g., components in furniture).^16,17^ Simultaneously, their environmental advantages, such as a microplastic-free biodegradable plastic alternative, should not be jeopardized.

In this work, we report the preparation and properties of carbon-fiber-reinforced plasticized WG-based materials. The reason to use both a reinforcement filler and a plasticizer (glycerol) is to obtain a combination of both strength and ductility/toughness in a step toward matching the mechanical properties of commonly used fossil-based plastics. The material was made by combining extrusion/compounding to produce well-dispersed and distributed carbon fiber composite filaments and injection molding to create products with the desired geometries. The experimental design allows direct implementation of the protocols in the industrial production of plastic composites, relying on extrusion/compounding and a further molding stage. We used a water-assisted extrusion method to process the material at a low temperature (significantly lower than, e.g., polyolefins) that, besides saving energy, also reduces the fiber breakage into smaller, less reinforcing species. The materials’ biodegradation was also assessed to validate the end-of-life of future gluten-reinforced bioplastics and ensure that the products could be considered within a cradle-to-grave frame.

## 2 Materials and Methods

### 2.1 Materials

Wheat gluten powder was supplied by Lantmännen Reppe AB, Sweden. It consisted of ca. 77 wt % protein (N × 5.7), 6 wt % starch, 1 wt % lipids, 6–8 wt % moisture, and 1 wt % ash. Glycerol (99%) was purchased from Thermo Fisher, Sweden. Carbon fibers were purchased from Jiangsu Horyen International Trade Co. Ltd., China, and were chopped for an average length of 2 mm. Chopped carbon fibers of 2 mm length are investigated here because previous work shows that 2 mm fiber length resulted in good mechanical performance^17^ (Figure S1, Supporting Information).

### 2.2 Preparation of the Gluten Composites

**Figure 1:**
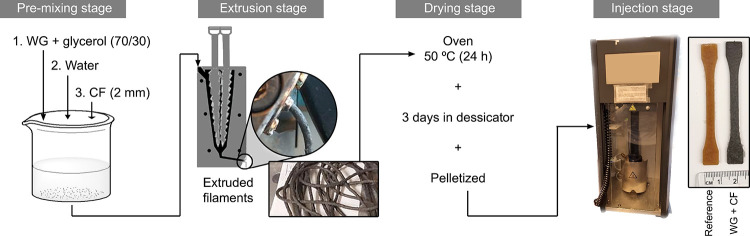
Illustration of the sample preparation using water-assisted extrusion in a mini-extruder and injection molding of the composites.

As-received wheat gluten powder (WG) was added to a beaker with glycerol to form a 30 wt % glycerol-plasticized matrix and mixed manually. The 30/70 w/w glycerol/gluten ratio was used based on previous studies.^17^ Millipore water (MQw) was gradually added to the formulation, equivalent to 20 wt % of the total mass, and the mixing process continued. The MQw was added due to its ability to decrease gluten-based formulations’ viscosity and increase fiber distribution and dispersion, including preserving the fiber length in the WG matrix.^17^ The 2 mm chopped carbon fibers (CF) were added and vigorously mixed until forming a homogeneous premixture (premixing stage, Figure 1). All WG composites contained 10 vol % of CF, corresponding to 13.4 wt %. The volume content of the fiber instead of the mass content is here used to compare CF’s reinforcing effects on the WG.

The above premixture was then compounded/extruded in a twin-screw mini extruder (Xplore instruments) at a constant rotation rate of 30 rpm (extrusion stage, Figure 1). Extrusion temperatures of 70, 90, and 100 °C were evaluated. The samples were dried at 50 °C for 24 h once extruded to remove the water and were then further dried in a desiccator containing silica gel for 3 days (drying stage, Figure 1). Glycerol/water-plasticized WG samples without fibers were also prepared as a reference. After that, the extruded filaments (reference and WG/CF composites) were chopped into ca. 0.5 cm long pellets and injected into a Hakke Mini-jet injection molder (Thermo Scientific). The samples were injected and molded into dumbbells on a small scale for further investigation (injection stage, Figure 1). Two injection molding parameters were tested: (i) 140 °C (cylinder and mold temperature) + injection pressure of 600 bar for 3 s + post pressure of 100 bar for 2 min, and (ii) same as before but 20 s at injection pressure + post pressure of 100 bar for 2 min. The parameters “i” were obtained from our previous work,^17^ and “ii” included here, aiming for further fiber alignment. The samples injected with 20 s injection time were labeled “N”. A summary of the different sample labels is shown in Table 1. Extruded filaments without being injected were also saved for determining the fiber structure in the extrudate related to the extrusion conditions used. A scheme of the preparation process is shown in Figure 1.

**Table 1:** Summary of Sample Composition, Processing Conditions, and Labels

sample	extrusion *T* (°C)	CF (vol %)	injection conditions
WG70	70	0	i
WG70N	70	0	ii
WG70CF10	70	10	i
WG70CF10N	70	10	ii
WG90	90	0	i
WG90N	90	0	ii
WG90CF10	90	10	i
WG100	100	0	ii
WG100CF10	100	10	i
WG100CF10N	100	10	ii

### 2.3 Mechanical Properties of the Biocomposites

Tensile tests of the injection molded dumbbell specimens (Type IV) were conducted in an Instron 5944 Universal Tensile Testing Machine with a 500 N load cell. The probes were conditioned at 23 °C and 50% RH for 72 h before the tests, according to ASTM D638-22, for materials with different rigidity. The dumbbells were strained at a crosshead speed of 10 mm min^–1^, and 4–5 replicates were used for each sample. Stress vs. strain profiles were obtained for each sample. In addition, Young’s modulus, maximum stress, and strain at break were calculated as the average with standard deviation.

Rheological properties of the mixtures with and without CF (premixing stage, Figure 1) were assessed to evaluate their viscoelastic behavior and suggest a processing window for the developed materials. The rheology was assessed via Dynamic Thermomechanical compression tests using a DMA850 (TA Instruments) with parallel plate geometry (diameter of 8 mm, gap of 2 mm). Temperature ramps were performed from 25 to 150 °C with a heating rate of 10 °C/min at a constant frequency (i.e., 1.0 Hz) and a strain amplitude of 0.01% within the linear viscoelastic range. In these tests, the storage (*E*′) and loss (*E*″) moduli and the loss tangent (tan δ = *E*″/*E*′) were collected from duplicates and reported as the average with standard deviation. Dynamic thermomechanical compression tests of protein-based composite blends have been typically used to select further processing conditions, such as injection molding.^18^

### 2.4 Biocomposite Morphology

The morphology of the tensile-fractured surfaces of the composite samples was examined in a field-emission scanning electron microscope FE-SEM (Hitachi S-4800) and a tabletop SEM (Hitachi TM100). Images of the specimen’s surface fracture were taken after the mechanical tests and on cryo-fractured samples. The specimens were immersed in liquid nitrogen for 2 min and fractured to obtain the cryo-fracture surface. The specimens were coated with a Pt/Pd alloy for 30 s by using an Agar high-resolution sputter coater (208RH).

The impact on the CF length after the intense compounding/extrusion (extrusion stage, Figure 1) was determined by observing the material in a light optical microscope (Inverted Laboratory Microscope Leica DM IL LED). A 1 g sample of the extruded filaments (before injection) was immersed in a beaker containing 30 mL MQw preadjusted to pH 10 (NaOH) for dissolving the gluten matrix. The filaments were stirred in the alkaline solution for 2 h, and aliquots of the resulting suspension were dropped on a glass slide and dried at 50 °C for 16 h. The fiber length and respective size distribution were obtained from 50 measurements using Imaje J.

### 2.5 Biodegradation and Assimilation of the Biocomposites

A soil degradation test assessed the biodegradation of the biocomposites and references, as previously described.^19^ Briefly, a 200 mg piece of material was buried in a composting medium (2:1 farmland: compost with 10% of vermiculite), according to ISO 20200:2004. The medium was kept humid throughout the test, with a 20 ± 5 °C temperature. The samples were carefully unearthed from the soil, and excess soil was removed without pressing the sample excessively. The photographed samples’ visual appearance was captured with time and buried in the same position afterward. The test finishes when the pieces of samples cannot be observed/rescued (fragments <1 mm).

**Figure 2:**
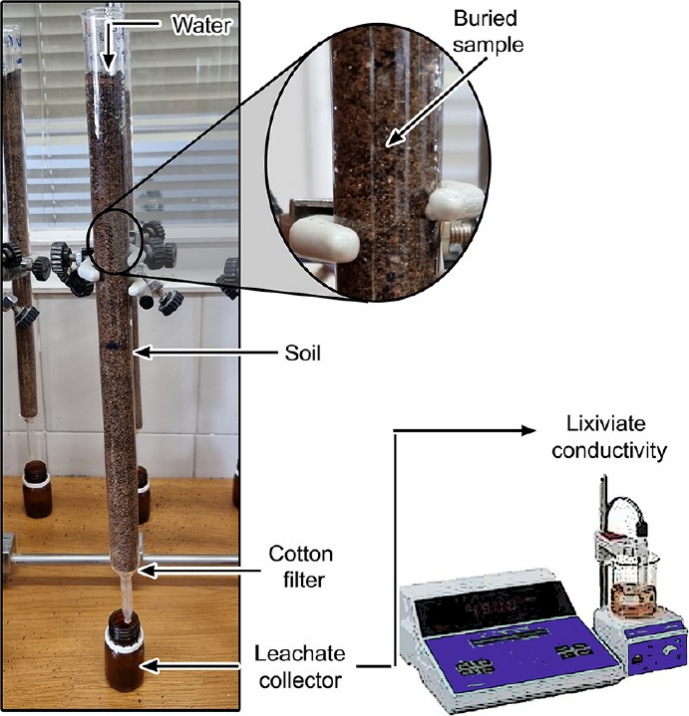
Experimental setup for the study of leachate (lixiviate) from the samples in soil.

The degradation process was also evaluated by studying the liquid being lixiviated from the samples, imitating regular irrigation conditions in agriculture.^20^ Briefly, 200 mg of the WG filaments after extrusion (extrusion stage, Figure 1) and WG/CF composites (injection molding stage, Figure 1) were buried in the soil used for the biodegradation above. The soil with the buried sample was kept in a glass buret with a cotton filter at the bottom, as shown in Figure 2. The systems were kept without light to simulate the natural process and for the microorganisms to act correctly. Twenty milliliters of water was poured from the top of the buret, equivalent to regular irrigation conditions in agriculture, i.e., 20 L water/m^2^.^19^ The lixiviated liquid was collected weekly, and its electric conductivity was measured using an EC-Meter BASIC 30 (Crison). The cumulative conductivity was used for constructing the lixiviation curves, and data adjustment was performed using the corrected conductivity by the reference soil (without a sample). It was considered that the sample was completely biodegraded when the leachate did not show significant differences with the reference soil for at least 3 consecutive days. The leachates were also evaluated by Fourier transform infrared spectroscopy (FTIR) to determine their physicochemical composition. For this, the samples were evaluated on a Hyperion 100 spectrometer (Bruker) with an ATR sensor. The measurements were obtained between 4000 and 400 cm^–1^ with an opening of 4 cm^–1^ and an acquisition of 100 scans.

A representative study of the impact of the biocomposites in the ecosystem was assessed using fast-growing grass seeds (*Cynodon dactylon*) on soil containing buried pieces of selected samples to ensure that the samples have no impact on the soil. The test was performed to preliminarily determine, at an early stage, if a future gluten-based bioplastic replacing synthetic counterparts lixiviates any component/substance that can hinder the regular growing activity of plants (represented here with fast-growing grass seeds) and to ensure a circular bioproduct. Approximately 200 mg of fragments of the selected samples were buried in a square parcel (10 × 10 cm^2^), and the grass seeds were evenly dispersed up to a density of 1 kg seeds/100 m^2^ (according to recommendations from the manufacturer). A control parcel with no samples buried was used as a reference.

## 3 . Results and Discussion

### 3.1 Carbon Fiber and Extruded Biocomposite Structure

**Figure 3:**
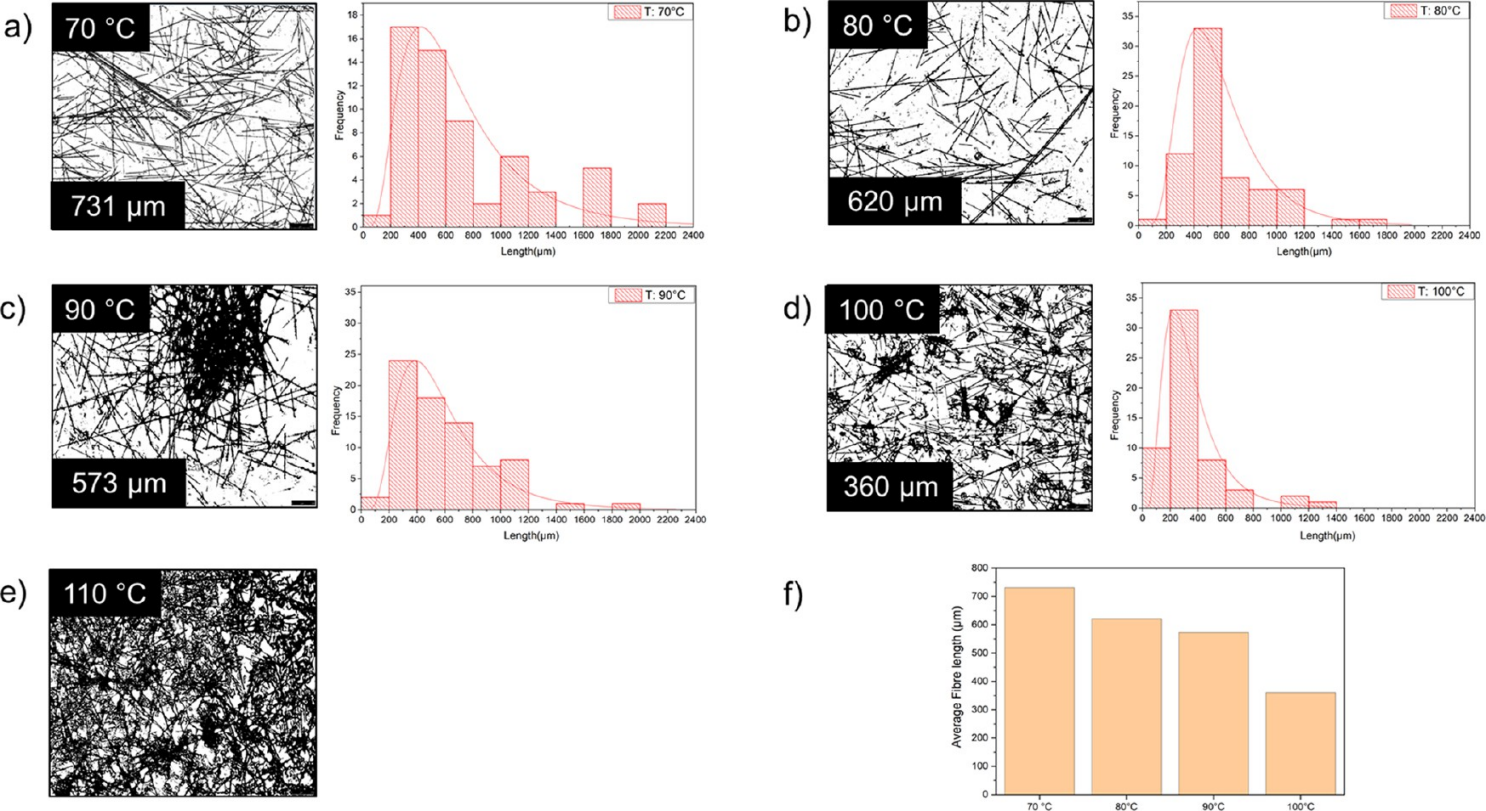
Optical microscope images of the carbon fibers (CF) and fiber length distribution after the gluten (WG) matrix dissolution from the extruded biocomposite at different extrusion temperatures: 70 (a), 80 (b), 90 (c), 100 (d), and 110 °C (e). The scale bar in the images is 250 μm. The average CF length at the different extrusion temperatures is shown in (f).

Figure 3 shows the carbon fiber (CF) size distribution after the extrusion of the filaments at different extrusion temperatures (extrusion stage, Figure 1). The dissolution of the gluten matrix revealed that the extrusion at the lowest temperature tested (70 °C) reduced the average length of the CF by ca. 64% compared to the original size (ca. 2 mm) (Figure 3a). The CF size distribution for the biocomposite extruded at 70 °C shown in Figure 3a was also the broadest among the different temperatures tested, consisting of large (>1600 μm) and medium size (<1000 μm) fibers. Increasing the extrusion temperature to 80 °C decreased the CF average size by ca. 70%, resulting in a sharper CF size distribution with no visible medium-size fibers and more distributed/dispersed fibers (Figure 3b). Figure 3c reveals that extrusion at 90 °C decreased the degree of dispersion of the CF compared with the 70 and 80 °C cases and resulted in large CF aggregates. Similar fiber sizes have been shown in the previous work on gluten-carbon fiber biocomposites processed using compression molding at 90 °C.^17^ The extrusion at 100 °C yielded smaller CF aggregates than those observed when extruding at 90 °C, resulting in the shortest CF average length measured and the most narrow size distribution (360 μm; Figure 3d). The extrusion at 110 °C resulted in a highly aggregated CF network, making it impossible to measure the CF sizes (Figure 3e). Hence, it is shown that heat- and shear-induced CF-gluten networks can greatly impact the mechanical properties of the injected samples and should be explored further in future studies.

The decrease in the average CF length with increased extrusion temperature originates from increased shear forces during extrusion due to gluten cross-linking at elevated temperatures.^5,8,21^ The increase in shear forces due to endogenous protein cross-linking can also improve CF distribution by influencing the aggregates’ dispersion. Notably, these formulations contain water, which increases swelling in the gluten protein network (acting as a plasticizer) and can also drive the formation of disulfide cross-links in gluten, especially at high temperatures.^22^ Thus, the biocomposites selected for postprocessing using injection molding were chosen among those extruded below 110 °C (70, 90, and 100 °C). In addition, at higher extrusion temperatures, especially near or above the boiling point of water (100 °C), the added water evaporates rapidly, leading to an increase in the viscosity of the gluten matrix, which increases shear forces and fiber breakage.

### 3.2 Properties of the Injection Molded Biocomposite

**Figure 4:**
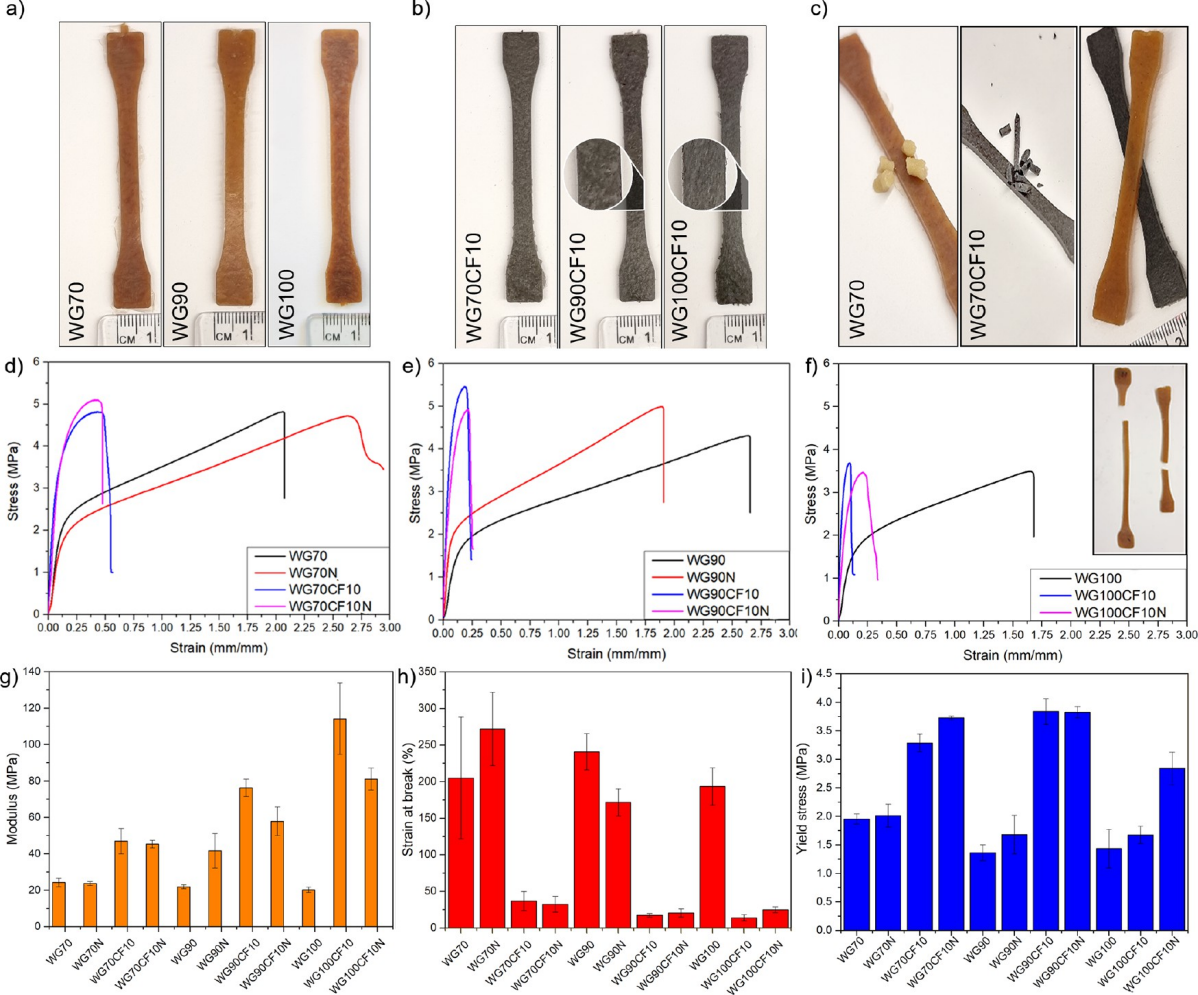
Dumbbell shapes of the injected wheat gluten (WG) specimens (a) and WG with 10 vol % carbon fiber (CF) (b). The injection molded samples were obtained from extruded WG mixtures at three temperatures (70, 90, and 100 °C). The inset in (b) shows high-magnification images of the dumbbell surface with apparent fiber alignment. Pellets from the extruded WG at 70 °C without and with CF and the respective injection molded specimens (c). Representative tensile stress–strain curves of the samples injection molded after extrusion at 70 (d), 90 (e), and 100 °C (f), Young’s modulus (g), strain at break (h), and yield strength (i) of the different samples. The inset in (f) shows representative WG100 (left) and WG100CF10 (right) specimens directly after the tensile fracture and 10 min later.

All extruded reference and filled gluten biocomposites could be injection molded into homogeneous dumbbell-shaped specimens at both injection molding conditions tested, independent of the extrusion temperature used to preprocess the materials (see Table 1 and Figure 4a,b). Examples of injection molded specimens from the pellet to the final molded shape, with and without CF, are shown in Figure 4c. It shows that the injection molded WG material without CF resulted in a darker caramel-colored sample than the extruded filaments (pelletized), which was due to Maillard reactions due to the high temperature in the mold (140 °C).^23,24^Figure 4b (insets) shows that the WG/CF dumbbell surface close to the neck area had flow lines at a ∼45° angle from the injection molding direction. These were more evident in the samples injection molded using pellets from the extrusion at 100 °C (WG100CF10) than those extruded at 70 °C (WG70CF10), as shown in Figure 4b. Yielding normally occurs by the onset of shear stresses 45° from the tensile direction.

The WG reference samples showed typical stress–strain curves for glycerol-plasticized WG with high strain at break and low elastic modulus, similar to previously obtained results (see Figure 4d–f).^17^ The elastic moduli (*E*) of the WG70, WG70N, WG90, and WG100 were similar and reached ca. 22 MPa (Figure 4g), demonstrating that the extrusion temperature and/or increase in the injection time (N samples) did not have an important role for the stiffness of the unreinforced WG samples. Therefore, the WG100N was not investigated further in this work. Overall, the WG systems without CF showed high strain at break (ca. 200%), with the samples WG70 and WG70N having larger standard deviations than WG90, WG90N, and WG100 (Figure 4h). The larger scatter of the low-temperature-processed WG70 sample could result from a less structured system than the preprocessed samples at higher temperatures and longer injection time. The inset in Figure 4f shows a representative specimen of WG100 directly after its tensile fracture and 10 min later. The WG without CF was highly stretchable and had a high elastic recovery after the 10 min (ca. 80%) compared to the filled WG matrix, which did not show a large deformation before fracture (inset in Figure 4f).

Adding the 10 vol % CF to the WG matrix resulted in a stiffer and stronger material, independent of the extrusion temperature and injection time (Figure 4d–f). Figure 4g shows that the extrusion temperature significantly affected the biocomposites’ modulus, increasing from 50 MPa (WG70CF10) to 120 MPa (WG100CF10). The results indicate a more effective stress transfer of the CF in the WG matrix when higher extrusion temperatures were used but also then yield shorter CF as shown in Figure 3d (a consequence of increasing protein aggregation). This is also an important feature for increasing the reinforcement effect of stiff fillers in soft matrixes.^25,26^ The increase in the injection time for the samples containing CF decreased Young’s modulus considerably for the samples that were extruded at higher temperatures (compare WG70CF10-WG70CF10N with WG100CF10-WG100CF10N, Figure 4g). The increase in the injection time coupled with the high mold temperature (140 °C) could result in a more denatured and aggregated WG, decreasing its ductility and promoting crack propagation during tensile load. The effect of the high temperature on the WG matrix was demonstrated by injection molding specimens from the same batch that had been contained for a longer time in the injection barrel (at 140 °C).

Figure S3 shows that the strain at the break decreased significantly with increasing residence time in the high-temperature barrel. The estimated residual time in the barrel between each injection was ca. 5 min. Thus, from Figure S3, it can be concluded that the residence time before the injection should be maximally 10 min to avoid a decrease in the WG material’s ductility. Note that the highest modulus obtained here (120 MPa) is comparable to that of low-density polyethylene, one of the most common polyolefins for applications in packaging, plastic bags, and plastic mulch films.^27^ It should be noted that the processing temperatures herein are about half of those typically used for LDPE manufacturing (reporting an embodied energy of 92 MJ/kg). Such a temperature decrease could result in energy savings equivalent to at least 6 kg CO_2_ emissions reduction (per kg of material produced), such as in previous reports where savings have been introduced to LDPE manufacturing using ultrahigh extrusion speeds.^28^ Furthermore, it is worth mentioning that this study revealed that the processing technique used to manufacture the materials also impacts the properties of the gluten biocomposite. Here, despite having similar fiber length and well-distributed CF in the gluten sample as compared to previous work using compression molding, the elastic modulus decreased ca. 50%.^17^ This is likely due to the extensive thermal effects induced in the gluten matrix during the high shear and temperature by the extrusion followed by injection molding (see Figure S3). Future work should increase the understanding of how these processing parameters affect the molecular structure of the protein toward improving their final properties and increasing their market competitiveness.

The effect of injection time and high temperature on the WG containing CF (WG100CF10 vs WG100CF10N) may be more important than in the reference specimens (WG100 vs WG100N) due to the higher thermal conductivity of the biocomposites. The strain at break for all filled samples was below 50% (Figure 4h), and the stress at yield was 3.7 MPa (Figure 4i). The low strain at break for the filled samples correlated well with their higher elastic modulus (more rigid systems). Figure S2 shows that the stress at break of the samples was 4–6 MPa. However, the stress at break data were more difficult to interpret because the differences between the samples with CF were insignificant. However, they had considerably lower stress at break than those without CF; for instance, compare the WG90 and WG90CF10 systems. It is, however, worth mentioning that the stress at break is not a critical mechanical property for the design of future bioplastic products (normally the material should not yield/deform plastically). The findings reveal that injection molding of pellets from extruded biocomposite matrix with only 10 vol % CF boosts the stiffness and strength of the bioplastics, here processed using mass production polymer processing techniques.

**Figure 5:**
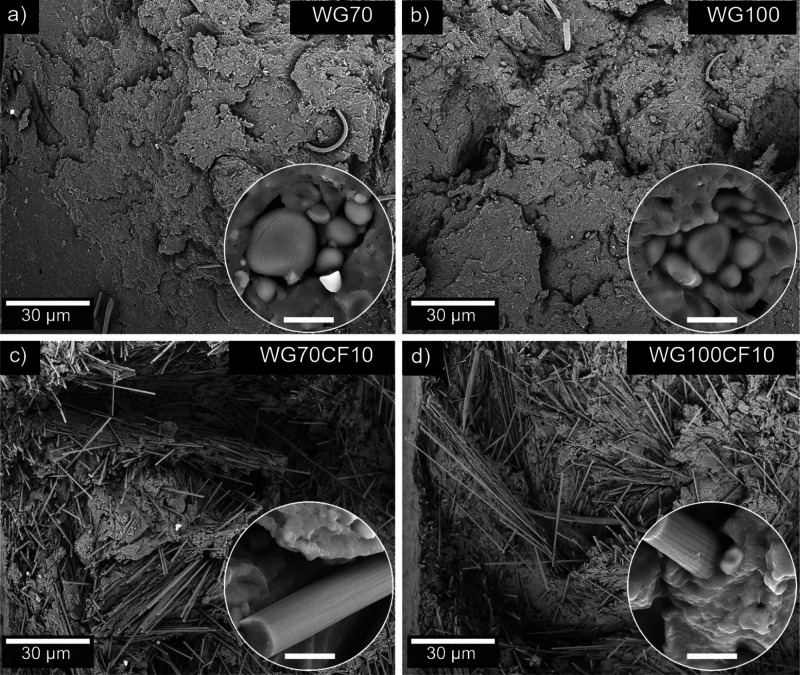
SEM images of the fracture surface after a tensile test of (a) WG70, (b) WG100, (c) WG70CF10, and (d) WG100CF10. The scale bar in the insets is 4 μm. The starch particles shown in the insets in (a) and (b) were spotted in some regions of the cross-section but did not describe the overall microstructure of the materials at high magnification.

The fracture surface after the tensile test of the WG70 and WG100 dumbbell specimens (unfilled) is shown in Figure 5a,b, respectively. Starch particles of ca. 20 μm in diameter were spotted in some regions in both WG samples, corresponding to traces left from the wheat starch extraction for producing the WG coproduct reported to be ca. 6–8 wt % (Figure 5a,b, insets).^29^ However, the starch aggregates spotted at high magnification from the WG sample injection molded using extruded pellets at 100 °C (WG100, Figure 5b) had defined welded zones between the particles, which were not observed for the WG70 sample. Figure S4a,b shows a higher magnification of the welded zones of these samples from the images shown in Figure 5a,b (insets). The result suggests that the presence of water in the WG raw material and an increase in the extrusion temperature (close to the gelatinization temperature of starch) could aid in forming a fused starch particle-WG matrix.^30^ A cohesive network between the starch particles and starch aggregate-WG matrix is favorable for increasing the homogeneity of the samples, despite starch being a minor component in the formulation. Thus, increasing the homogeneity of the entire formulation reduces crack formation and propagation and decreases the number of voids at the interface (see Figure 5a,b). The results agree with samples WG90 and WG100 having a strain at the break with lower standard deviations compared to WG70 (Figure 4h).

Figure 5c,d shows the tensile fracture surfaces of the WG70CF10 and WG100CF10 biocomposites, with several pulled-out CF fibers at the fracture surface. The appearance of the surface fracture suggests that the fracture occurred at shear planes 45° to the stress plane, which is a clear indication of a ductile fracture. In addition, most of the CF observed in the SEM images were pulled out at a certain angle, suggesting a pre-orientation of the fibers due to the injection molding process (refer to Figures 4b and 5c,d). Figure 5c,d (insets) and Figures S4c,d show good interfacial bonding between the CF and the WG matrix, which agrees with the improved mechanical properties of the biocomposites by adding only 10 vol % carbon fibers.

**Figure 6:**
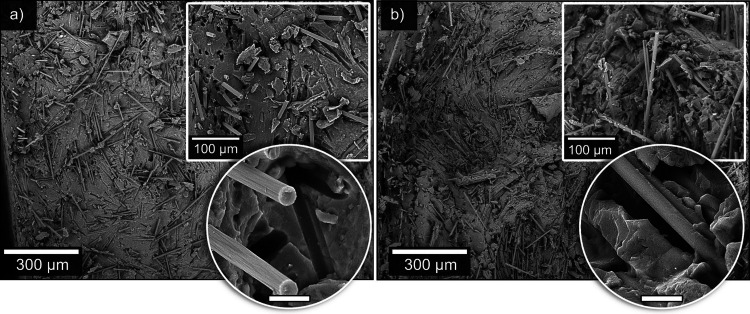
SEM images of the cryo-fractured surfaces of the injection molded biocomposites: WG70CF10 (a) and WG100CF10 (b). The images were taken in the neck area of the injection-molded dumbbell specimens. The insets show higher magnification images of the surface. The scale bar in the inset is 10 μm.

The microstructure of the biocomposites was also studied on cryo-fractured WG70CF10 and WG100CF10 specimens to evaluate the CF distribution/dispersion in the WG matrix with minimal plastic deformation (Figure 6). The SEM images represent the cross-section at the dumbbell neck. The microstructure of the WG70CF10 shows carbon fibers in random orientation in the surface plane, i.e., perpendicular to the injection direction, which were pulled out at different angles (Figure 6a). A higher magnification image of WG70CF10 shows holes and marks from fully pulled-out carbon fibers during cryo-fracturing. The WG100CF10 sample had a random CF orientation similar to that of WG70CF10 (Figure 6b). However, fewer holes were observed in the WG100CF10 sample (extruded at 100 °C), suggesting stronger interactions formed between the CF and WG matrix compared to WG70CF10. Higher magnification of the WG100CF10 reveals several WG matrix fragments attached to the CF or the fibers embedded inside the protein matrix (Figure 6b). This agrees with the WG100CF10 samples having a 58% higher Young modulus than the WG70CF10 sample (Figure 4g). The improved interfacial adhesion between CF and WG with the increased extrusion temperature is possibly due to the higher tackiness of the glycerol-plasticized WG at higher temperatures.^31^

**Figure 7:**
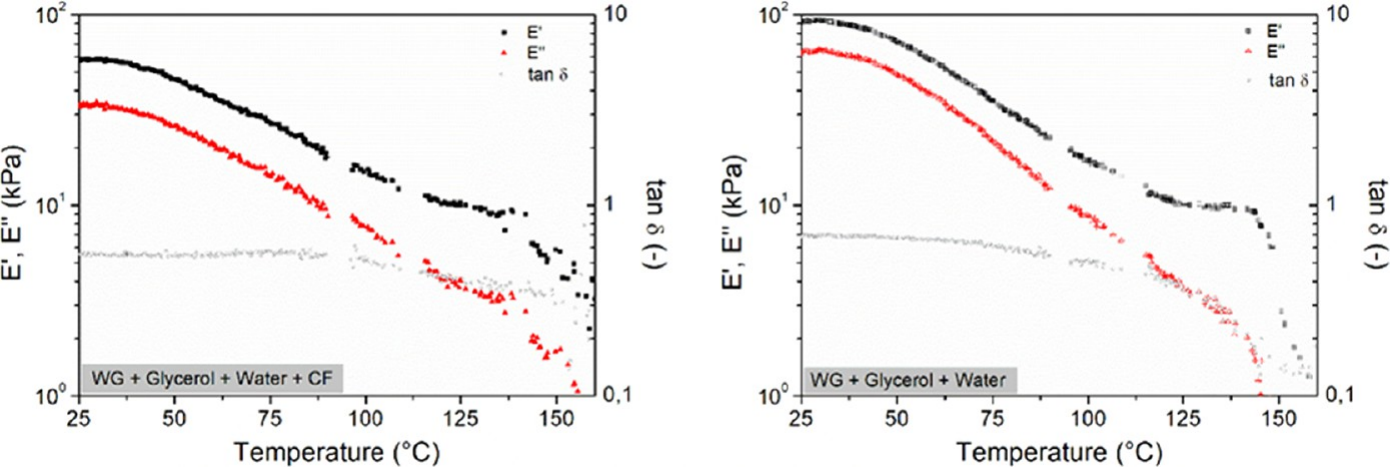
Temperature ramp profile of WG, glycerol, and MQw blends before extrusion with and without 10 vol % CF.

The rheological behavior of the WG blends (no thermal processing) with and without CF was evaluated through the temperature ramps, as shown in Figure 7. Both blends had a similar profile where 4 stages were observed: (i) an initial stage where *E*′ and *E*″ remain constant with the temperature, (ii) followed by a significant decay of both moduli (glass transition region), (iii) that leads to a new stage where *E*′ remains constant, while *E*″ decays (stiffening of the system), and (iv) finally a sharp drop in both moduli. These stages were also observed in previous studies.^32,33^ The different stages were well resolved in the fiber-free WG. These appeared at 25–30, 30–120, 120–145, and 145–160 °C for the WG reference and at 25–40, 40–115, 115–140, and 140–160 °C for the reinforced WG. The lower moduli in the WG mixture with CF are ascribed to more voids/trapped air due to the presence of the fibers before processing these, which leads to a less dense structure than the WG without CF. This is also corroborated by the fact that the difference in *E*′ and *E*″ values between CF-loaded and non-CF-loaded samples decreases steadily with increasing temperature until it disappears above 115 °C. This reduction in viscoelastic properties found after adding CF would facilitate further biocomposite processing, such as injection molding below this temperature.

Figure 7 shows that the tan δ remained constant until *E*″ decreased faster than *E*′, reducing the value of the tangent. This decay occurs earlier in the WG without CF (65 vs 90 °C), as seen in Figure 7. This behavior indicates a higher heat distortion temperature in the presence of CF.

From the rheological results, it is possible to suggest operating temperatures for implementing these recipes in polymer processing. The temperature in the extruder must be less than that at which the tan δ decays, i.e., 65 and 90 °C for WG without and with CF, respectively, to avoid excessive cross-linking reactions in the WG matrix before entering the mold. The results agree with the WG specimens becoming stiffer with a longer residence time in the injection molding barrel at temperatures above 100 °C (see Figure S2). In addition, mixing will be favored at temperatures close to the tan δ decay limit since the stiffness/rigidity is lower, requiring less mechanical energy for the processing. These results are consistent with those obtained in the previous sections, where CF agglomerates were observed with an extrusion temperature of 90 °C and higher, which correlates to when the material begins to harden and makes the dispersion of filler more difficult. As for the injection molding process, the necessary conditions should be established in the molding to favor the thermosetting of the systems, promoting the development of cross-links that allow the material to be given its structure and final properties. In this sense, the temperature should be in the third stage of the temperature ramp, i.e., 120–145 and 115–140 for the reference and CF systems, respectively (Figure 7), where *E*′ remains constant while *E*″ decays. This curing/hardening will be greater as the mold temperature is established closer to the upper limit. However, it is important not to exceed this limit and not to compromise the structure (if the temperature increases, *E*′ drops rapidly).

### 3.3 Biocomposite’s End-of-Life

**Figure 8:**
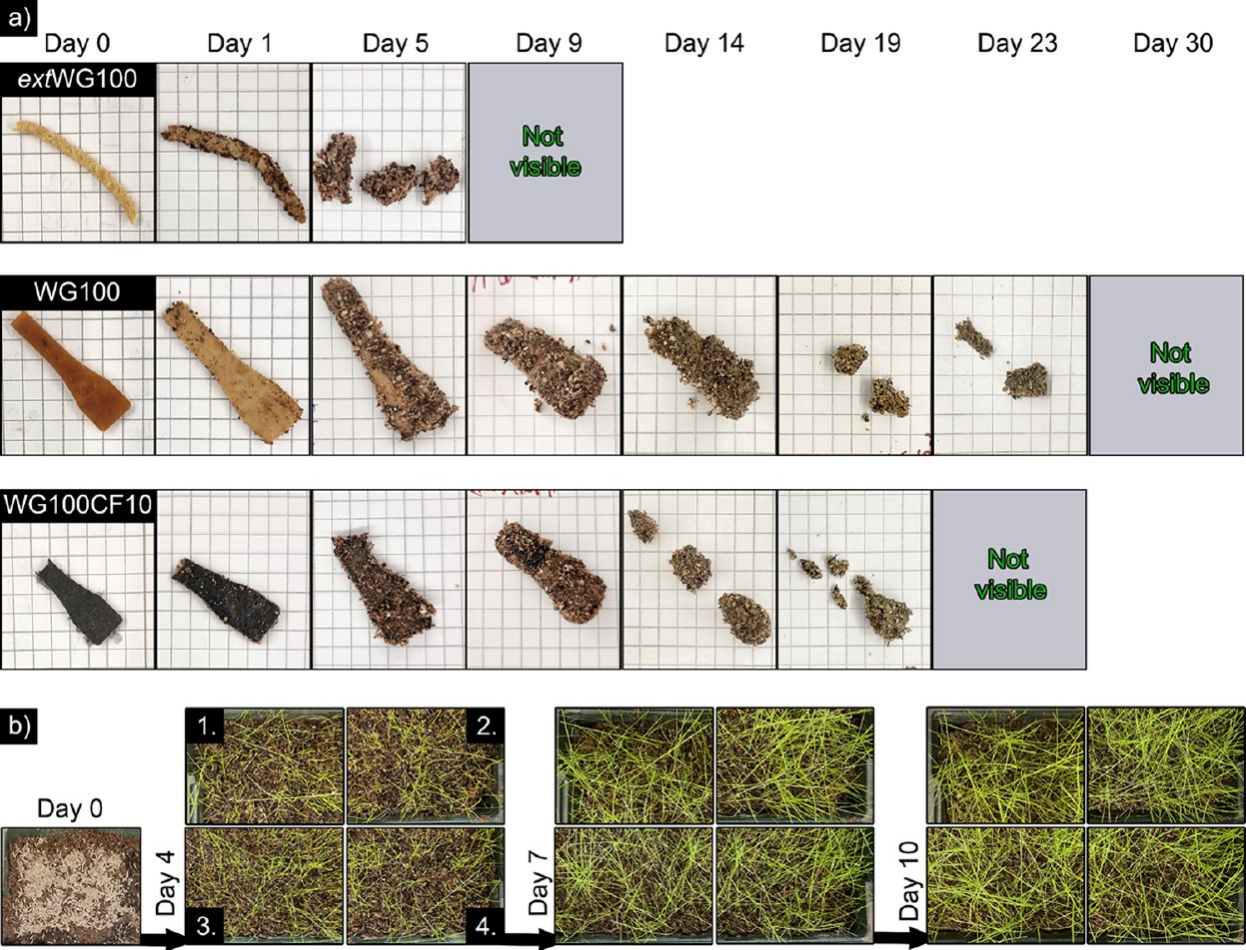
Biodegradability of the extruded WG filament at 100 °C (extWG100), WG extruded at 100 °C and injection molded (WG100), and WG/10 vol % CF extruded at 100 °C and injection molded (WG100CF10) (a). The squares in “a” correspond to 0.5 cm × 0.5 cm. Bioassimilation of the biocomposites buried in soil with grass seeds on top after different periods (b). The different parcels correspond to the control soil (b1.), extWG100 (b2.), WG100 (b3.), and WG100CF10 (b4.).

Figure 8a shows the biodegradation of the extruded WG filament at 100 °C, WG100 (extruded at 100 °C, pelletized, and injection molded (Table 1)), and WG100CF10 (same as WG100 but containing 10 vol % CF). The extruded WG filament started to fragmentate on day 5 and was fully biodegraded after 9 days. On the contrary, the WG100 sample began to change shape and fracture after 14–19 days and biodegrade after day 30 (Figure 8a). The change in color from day 0 to day 1 for WG100 is due to the matrix swelling in the moist soil used for biodegradation. The sample containing 10 vol % CF (WG100CF10) degraded slightly faster than the WG100, possibly due to voids between the CF and WG matrix facilitating the microorganism’s interaction with the protein matrix (Figure 8a). The sample’s biodegradation behavior aligns with previous reports on thermally processed protein-based bioplastics.^34^ Thus, it was demonstrated here that gluten could be used as a matrix to produce single-use thermoprocessed microplastic-free items with rapid biodegradability. According to ASTM D64000, all samples could be considered biodegradable and compostable, as they degraded before 90 days. However, according to the standard, an assessment must ensure that no toxic reagents are released into the soil.

Figure 8b shows the soil parcels where fragments of the extruded WG filament, WG100, and WG100CF10, were buried and covered by fast-growing grass seeds to evaluate the bioassimilation of postconsumed gluten bioplastics and ensure no toxic reagents released into the soil. All parcels showed fast germination of the grass seeds (day 4) and long and healthy grass leaves after 10 days (Figure 8b). No exclusion zone for growing seeds was observed around the areas where the bioplastics were buried, and the leaves were similar to those of the control sample (Figure 8b1). The grass yield was also similar to that of the control parcel (Figure 8b1) and those containing gluten bioplastics (Figure 8b2–b4). The results indicate that the materials are innocuous for germinating and growing seeds, strengthening the fact that sustainable reinforced-gluten bioplastics are biodegradable and safe for nature if disposed of. Therefore, it has been demonstrated that no toxic substance for soil or plant growth is released during the biodegradation of these samples, complying with ASTM D64000. Even if the amount of fiber added was low and had no evident impact on the soil, it is important to consider its presence in the end-of-life scenario of these materials. However, in the same way as biodegradable glass fibers have been developed in recent literature,^35^ it is not unlikely that biodegradable carbon fibers will be available in the future. Here, studies addressing a sustainable engineering process report the commercial production of biobased carbon fibers based on cellulose and lignin,^36^ which is the first step to designing future biodegradable CF.

**Figure 9:**
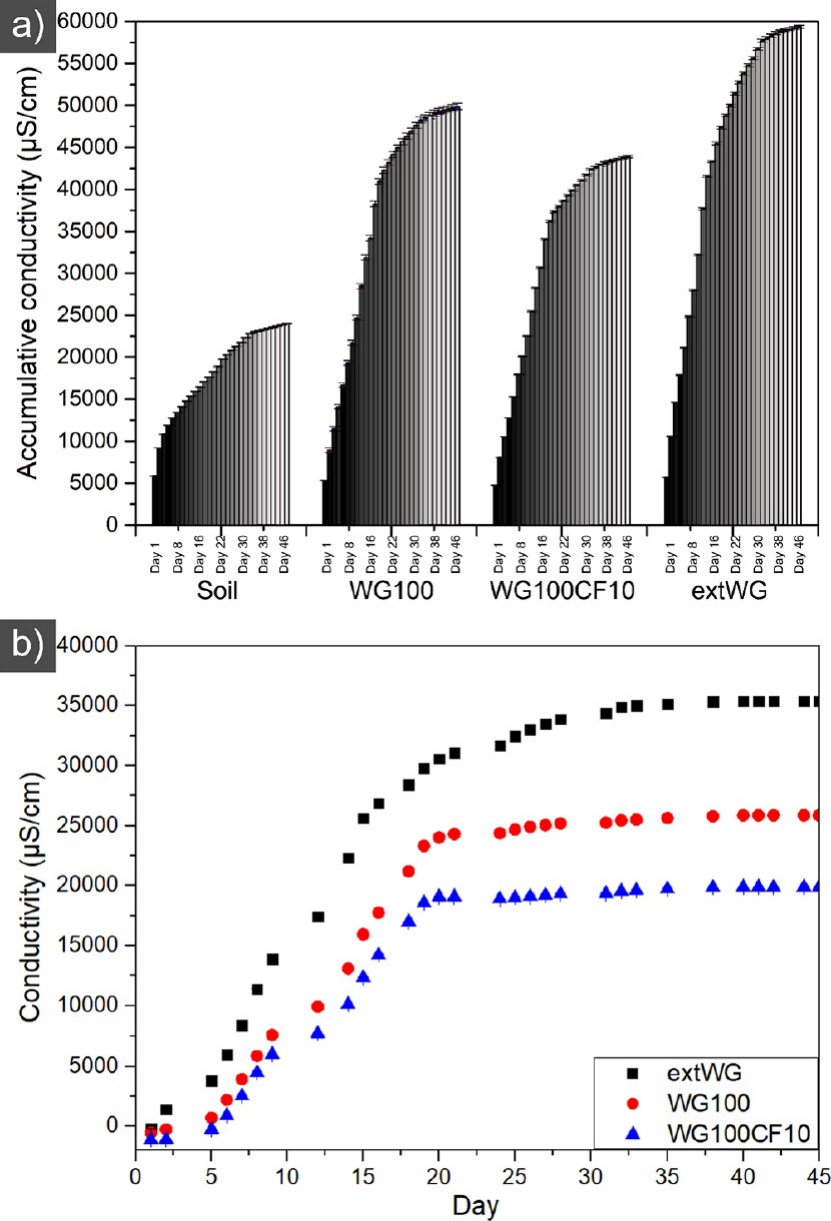
Accumulative conductivity of the water lixiviated from the soil containing the WG100, WG100CF10, and extWG (a) and corrected conductivity after irrigation periods (b).

Figure 9a shows that the soil samples with the gluten-based biocomposites’ accumulative conductivity almost doubled compared to the reference soil. It should be mentioned that the amount of material added was only 200 mg vs ca. 500 g of soil used to fill the buret (Figure 2). The increase in conductivity is due to nutrients and short polypeptide chains being lixiviated from the protein material with the irrigated water. The results follow reports on protein-based materials loaded with nutrients for the soil.^12,19,37^Figure 9b shows the corrected conductivity of the biocomposites relative to that of the reference soil release. The WG filament extruded at 100 °C started to yield an increase in soil conductivity after 2 days, showing the highest release slope/rate. The release from the WG100 and WG100CF10 were similar, with the WG100 having a higher release due to the latter containing 10 vol % of CF (Figure 9b). The results agree with the biodegradation data, showing that the extruded WG had the highest degraded rate (Figure 8a). The lixiviation time is normally slower than the biodegradation time because it takes a longer time (and more water) to leach out all of the low-molecular species/nutrients from the materials. Nevertheless, this is an interesting property for the slow release of nutrients to plants or soil, which is only possible by using polymer matrixes that degrade into innocuous molecules for the environment or do not produce microplastics.

**Figure 10:**
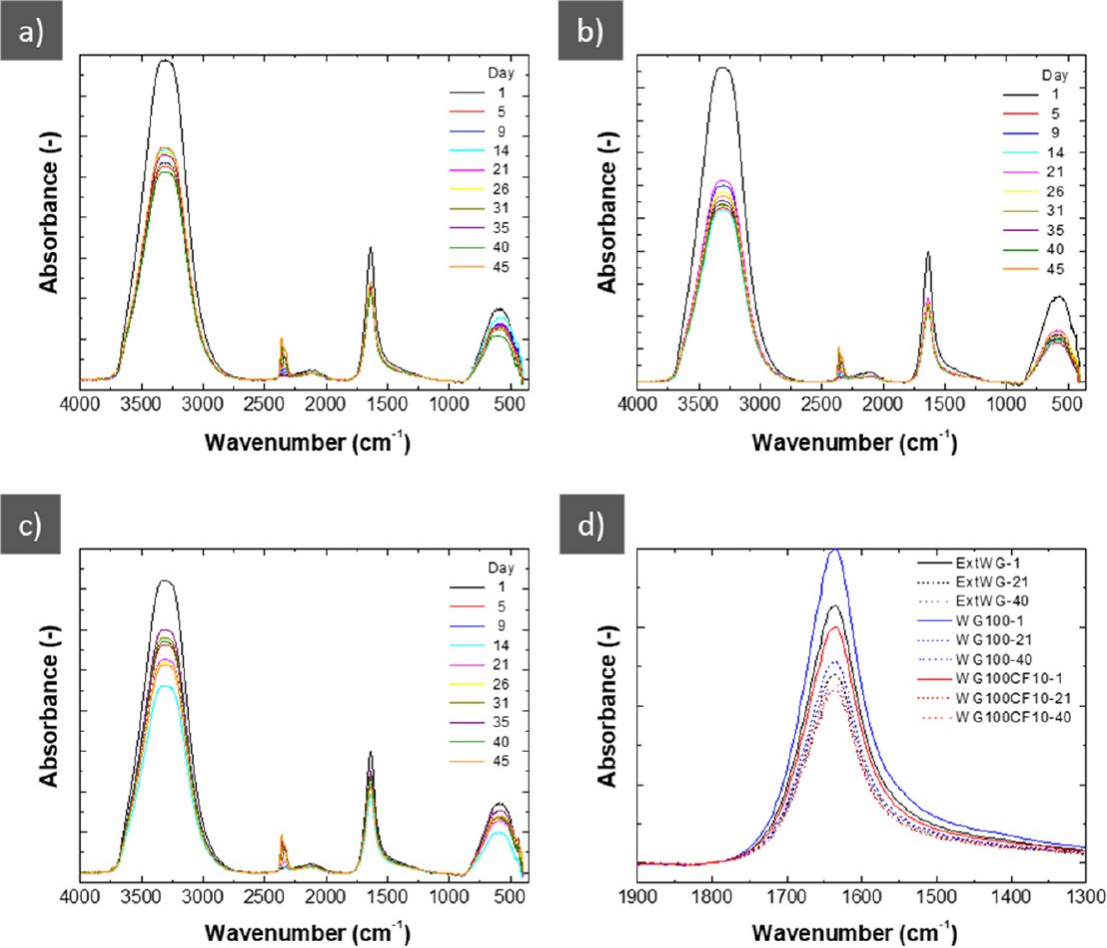
FTIR profiles of the water lixiviated from the soil containing extWG (a), WG100 (b), and WG100CF10 (c). In addition, a comparative of the amide peak of each sample (d).

Figure 10 shows the FTIR profiles of the leached water during the assimilation assays. All systems present a large absorbance in the 3750–2750 cm^–1^ band, corresponding to the O–H bonds of water, since it is the main component of these leachates. However, the most interesting peak in these spectra is observed at 1850–1550 cm^–1^, corresponding to amide I. During biodegradation, protein chains shorten, causing the amide I peak to lose intensity in FTIR.^38^Figure 10 shows that the amide I peak decreased gradually with increased biodegradation time. It should be noted that the WG samples without CF (extWG and injection molded WG100, Figure 10a,b, respectively) had more soluble proteins being leached out already on day 1. The result is consistent with the soil degradation results, showing that the extruded WG100 degraded faster than the other samples (Figure 8). However, WG100CF10 contained less protein, which could also decrease the amide I peak (Figure 10c).

## 4 Conclusions

Protein-based biocomposites with improved mechanical properties and rapid biodegradation and bioassimilation were manufactured using carbon fibers embedded in the wheat gluten matrix. The biocomposites were prepared by mixing the components and extruding them at different temperatures, leading to filaments that were subsequently pelletized and injection molded. The mixing/extrusion temperature impacted the CF size distribution and dispersion within the gluten matrix, and the CF length decreased with an increase in the extrusion temperature. Moreover, it was possible to process the materials using temperatures less than half of those used for synthetic polyolefins. In addition, the sole addition of 10 vol % of CF increased the elastic modulus up to 4 times and doubled the yield strength of the injection molded materials. The final mechanical properties were a consequence of the presence of the fiber but also affected by the strong glycerol-induced plasticization of the gluten matrix, residual partly fused starch particles contained in the matrix, low-temperature processing/high shear forces during extrusion, and the partial alignment of the CF during the injection molding. Despite the extensive thermal processing of the reinforced gluten-CF biocomposites (extrusion + injection molding), the materials showed rapid biodegradation (less than 30 days), yielding leachate of microplastic-free oligomers/molecules that enriched the soil. The materials are also nontoxic to the soil despite their fast degradation and carbon fiber content, as demonstrated by the growth of grass seeds. This is a relevant factor in assessing the safety of these materials, even if they are misplaced in nature by users or have wrong waste management policies after their end-of-life. The results show the advantage of using gluten for future manufacturing of consumer products that are safe, microplastic-free, and environmentally friendly from the raw material to the postconsumption stage.

## Supporting Information Available

SEM image of the chopped carbon fibers; stress at break of the different samples; strain–stress curves of the reference WG formulation; and high-magnification SEM images of the tensile fracture surface of the samples (PDF)
