# Supplementary material for: Biodegradable Fiber-Reinforced Gluten Biocomposites for Replacement of Fossil-Based Plastics
Source: ACS Omega. 2023 Dec 1;9(1):1341–51. doi: 10.1021/acsomega.3c07711 (PMC10785611; doi:10.1021/acsomega.3c07711)
Supplement: Supplementary file 1 — ao3c07711_si_001.pdf [file ao3c07711_si_001.pdf]

Supporting Information

Biodegradable fiber-reinforced gluten biocomposites for replacement of fossil-based plastics

Antonio J. Capezza[a*], Mercedes Bettelli,[a] Xinfeng Wei,[a] Mercedes Jiménez-Rosado,[b] Antonio Guerrero,[b] Mikael Hedenqvist[a*]

Author's affiliations:

[a]Department of Fibre and Polymer Technology, KTH Royal Institute of Technology, Teknikringen 56, SE-100 44 Stockholm, Sweden

[b]Department of Chemical Engineering, Universidad de Sevilla, 41012 Sevilla, Spain

*Corresponding authors: ajcv@kth.se ,mikaelhe@kth.se

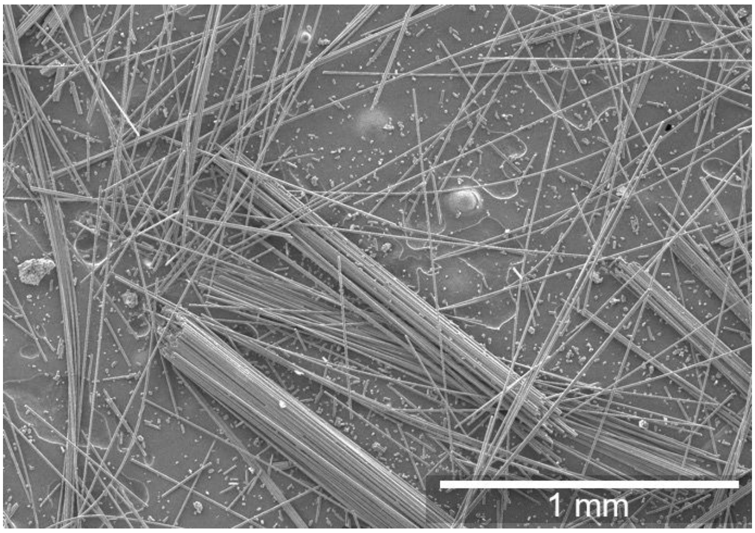

Figure S1. SEM image of the chopped carbon fibers with an average length of 2 mm.

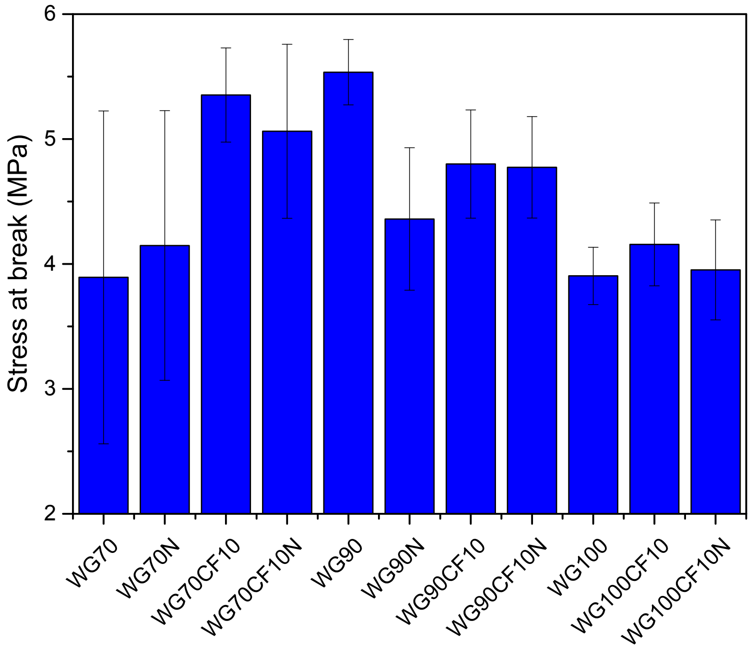

Figure S2. Stress at break of the different filled and unfilled samples.

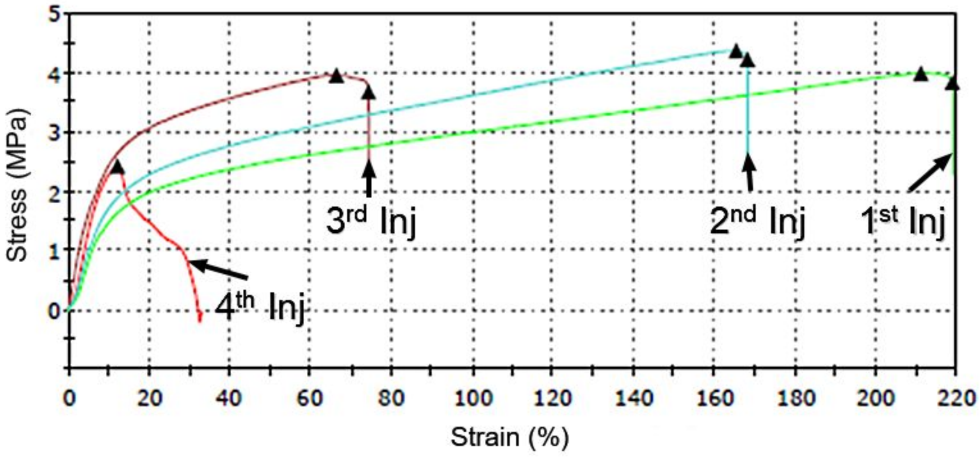

Figure S3. Strain-stress curves of the reference WG formulation (no carbon fibers). The 1st to 4th indicates the order in which the material was injected using the same extruded batch material in the injection cylinder.

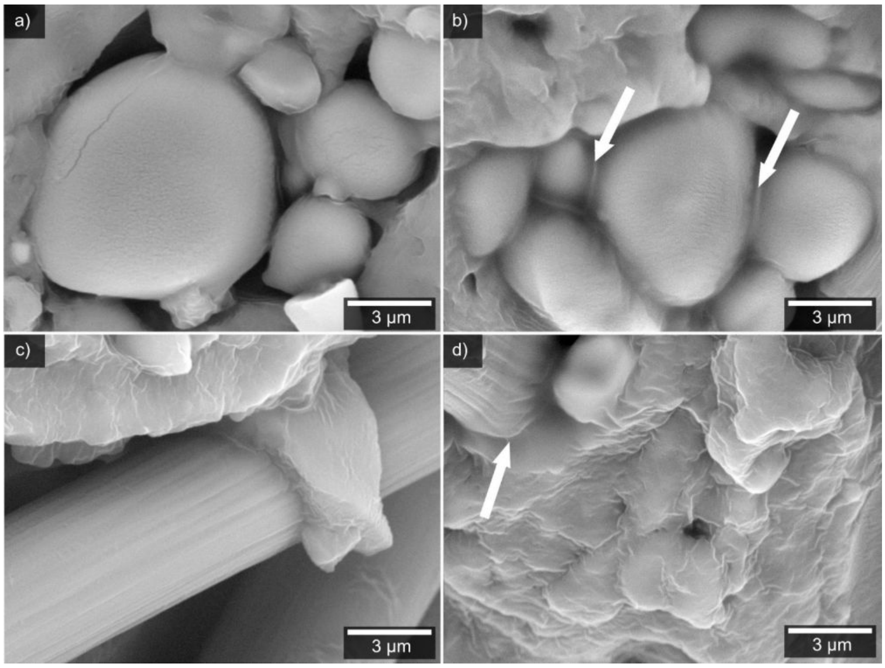

Figure S4. High-magnification SEM images of the tensile fracture surface of the WG70 (a), WG100 (b), WG70CF (c), and WG100CF (d). The arrows in (b) point to weld lines between starch particles and in (d) the interface region between a carbon fiber (CF) and the WG matrix.
